# Trends and Collaborations in Mesenchymal Stem Cell Research Applied to Animal Models of Osteoarthritis: A Bibliometric Analysis (2015–2024)

**DOI:** 10.1155/vmi/8110208

**Published:** 2025-11-20

**Authors:** Jorge U. Carmona, Luis H. Carmona-Ramírez, Catalina López

**Affiliations:** ^1^Department of Animal Health, Regenerative Therapy Research Group, University of Caldas, Manizales, Colombia; ^2^EFE Research Group, Catholic University of Manizales, Manizales, Colombia; ^3^Department of Animal Health, Veterinary Clinical Pathology Research Group, University of Caldas, Manizales, Colombia

**Keywords:** animal models, mesenchymal stem cells, scientometric analysis, translational research

## Abstract

**Background and Aims:**

There is increasing interest in the clinical use and experimental evaluation of mesenchymal stem cells in animal models of osteoarthritis (OA); however, no bibliometric analysis has been published on this topic.

**Methods:**

A bibliometric analysis was performed using the bibliometrix R package by analyzing the documents registered in the WOS database from 2015 to 2024. The registers were evaluated according to overview, sources, authors, documents, words, trend topics, clustering, and conceptual intellectual and social structures.

**Results:**

The articles were mainly published in Stem Cell Research and Therapy, OA and Cartilage, International Journal of Molecular Sciences, Scientific Reports, American Journal of Sports Medicine, Journal of Orthopedic Research, Stem Cells International, Arthritis Research and Therapy, and Biomaterials. The most productive institutions were Zhejiang University, Shanghai Jiao Tong University, and Peking University, and the most productive countries were China, the USA, and Korea. The most frequently used keywords were OA, mesenchymal stem cells, and cartilage. The trending topics in this area are cartilage repair, exosomes, and extracellular vesicles. The collaborative network of authors, institutions, and countries is led by Chinese authors and institutions.

**Conclusion:**

This bibliometric analysis reveals a significant increase in research on mesenchymal stem cells for OA, primarily led by Chinese institutions. Key topics include cartilage repair and exosomes, highlighting a collaborative global network that is shaping the future of therapeutic strategies in this field.

## 1. Introduction

Osteoarthritis (OA) is a chronic debilitating disease that affects not only cartilage integrity, but also subchondral bone and intra- and periarticular soft tissues [1, 2]. It is the most common joint disease in humans and domestic animals. In humans over the age of 30, the disease affects 15% of the world's population [3], while in dogs over the age of 1, the condition affects 20% [4], and in dogs over the age of 8, OA can be diagnosed in the 42.3% of the population [5]. On the other hand, in horses, OA is responsible for 60% of lameness clinical cases [6].

In general, the pathophysiological mechanisms of OA and even the clinical symptomatology are similar across species. For these reasons, animal models of naturally occurring OA or experimentally induced OA have been used as translational tools to understand this joint disease in humans [7, 8]. Currently, there are several experimental and clinical treatments for the management of OA in humans and animals. However, from a practical point of view, this disease remains an untreatable condition that in many cases could end in a total joint replacement [9].

Regenerative medicine has emerged as a multidisciplinary field of research aimed at restoring tissues and organs affected by diseases of various origins, including traumatic, degenerative, inflammatory, and congenital, among others [10, 11]. Currently, there are several clinical and experimental regenerative medicine-based therapeutic approaches for the treatment of OA in humans [12–14] and animals [15–17], including the use of stem cells, gene therapy, advanced biomaterials, platelet-rich plasma, autologous conditioned serum, and autologous protein solution, among others [18].

The use of stem cells for the treatment of OA in humans remains a prolific area of research [19–21], continually supported by translational studies in animal models [2, 22–25]. It is important to clarify that the term “stem cells” serves as an umbrella that encompasses various types, including fetal (such as embryonic stem cells), adult (such as mesenchymal stem cells [MSCs]), and induced pluripotent stem cells (iPSCs) [26–28].

MSCs have shown promise in the treatment of OA in several animal models, including both naturally occurring and experimentally induced OA [2, 29]. MSCs are found in a variety of tissues including adipose tissue, bone marrow, umbilical cord tissue, dental pulp, peripheral blood, and synovial membrane and periosteum. These multipotent cells can self-renew and differentiate into various cells of the mesenchymal lineage, including chondrocytes, osteoblasts, adipocytes, and myocytes [26–28, 30].

To the authors' knowledge, no published studies have evaluated the bibliometric impact of the scientific literature on the experimental and clinical use of MSCs in animal models of OA. Bibliometric landscape analysis is a valuable computational tool for assessing the temporal evolution of a specific scientific topic or body of knowledge in a given field. This technique examines the number and impact of publications, trends over time, key authors, and influential papers, providing insight into the development and influence of research topics [31, 32].

The study aimed to perform a bibliometric analysis of the scientific literature published over the last 10 years on the evaluation of the therapeutic potential of MSCs in animal models of OA. This analysis seeks to determine how this research topic is influenced by authors, institutions, and countries, and how their research agendas have affected the use of keywords, trending topics, topic clustering, conceptual structures, and social structures related to the topic.

## 2. Materials and Methods

This bibliometric study was conducted between July and September 2024. Ethical approval was not required because this study did not involve human subjects or live organisms.

### 2.1. Database Selection, Keyword Search Structure, and Search Refine

Web of Science (WOS) (Clarivate Analytics, London, UK) was selected for this bibliometric analysis. This database includes more than 12,000 journals published in 45 languages in the various fields of science and allows bibliometric analysis, including citation counts, citation tracking, and H-index calculations [33].

The following search equation was used for this bibliometric analysis:

(“MSCs”) AND (“animal model” OR rat OR mouse OR rabbit OR dog OR canine OR horse OR equine OR “nonhuman”) AND (“OA” OR “degenerative joint disease”).

Once the initial results were obtained, they were filtered by year (2015–2024) and language (English only). Furthermore, document types such as reviews, editorials, book chapters, notes, retracted papers, conference papers, short surveys, letters, and erratum were excluded to include only articles.

### 2.2. Bibliometric Analysis and Visualization Tools

The data were exported from the WOS database in plain text file format and analyzed using the R package bibliometrix (Version 4.4.0). The results were visualized using the biblioshiny web application [32]. The analysis was performed using the general menu of the app, which includes features for overview, sources, authors (including affiliations and countries), documents, keywords, trending topics, clustering, and conceptual, intellectual and, social structures.

## 3. Results

**Figure 1:**
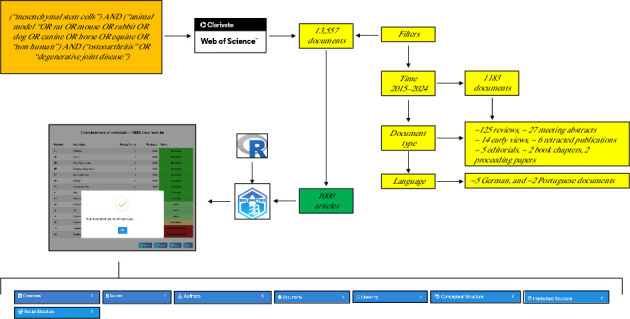
Design of the study and register workflow.

The WOS database initially yielded 1357 documents. Of these, 1183 were published within the study period (2015–2024). Of these 1183 records, there were 125 review articles, 27 meeting abstracts, 14 early views, 6 retracted publications, 5 editorials, 2 book chapters, and 2 proceedings papers, for a total of 1007 documents. From this total, 5 documents in German and 2 in Portuguese were excluded, resulting in a final validated count of 1000 articles.  Figure 1 summarizes the design of the study and register workflow.

### 3.1. Overview

During the observation period of this bibliometric analysis, 1000 research articles on this topic were published in 336 journals, representing an annual growth rate of 4.08%. The average age of the documents was 4.11 years, with an average of 25.14 citations per document. About 31,239 references were cited, and 5342 authors used 2124 keywords in these documents.  Table 1 shows the chronological evolution in the production of records per year, as well as the average of citations per year for these documents.

### 3.2. Sources (Journals)

**Figure 2:**
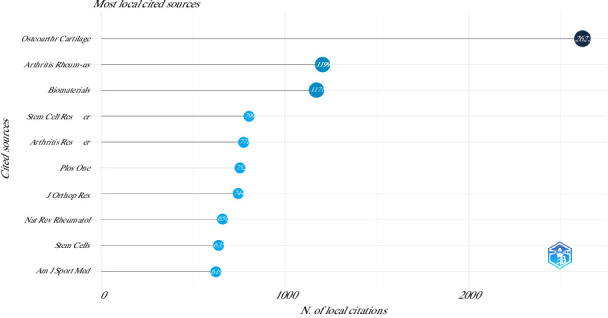
Top 10 most local cited sources on the use of mesenchymal stem cells in animal models of osteoarthritis (2015–2024).

Stem Cell Research and Therapy (37 articles), OA and Cartilage (31 articles), International Journal of Molecular Sciences (24 articles), Scientific Reports (23 articles), American Journal of Sports Medicine (21 articles), Frontiers in Veterinary Science (20 articles), Journal of Orthopedic Research (18 articles), Stem Cells International (17 articles), Arthritis Research and Therapy (16 articles), and Biomaterials (16 articles) were the top 10 most relevant sources. However, the top 10 most cited journals were OA and Cartilage, Arthritis and Rheumatism (USA), Biomaterials, Stem Cell Research & Therapy, Arthritis Research Therapy, Plos One, Journal of Orthopedic Research, Nature Reviews Rheumatology, Stem Cells, and American Journal of Sports Medicine (Figure 2).

**Figure 3:**
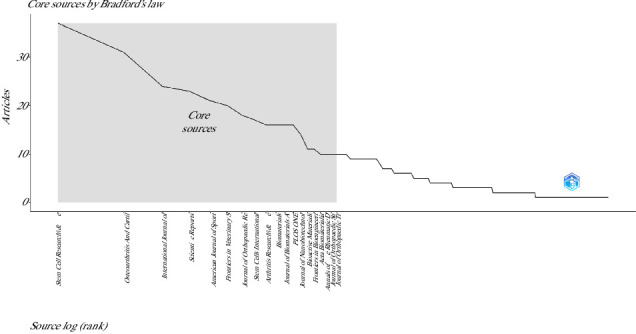
Distribution of the core group of highly productive sources on the use of mesenchymal stem cells in animal models of osteoarthritis (2015–2024).

According to Bradford's Law [34], 19 sources were classified as core journals in this research topic (Figure 3). In terms of the local impact of the journals as measured by the H-index, the top 10 journals on this topic were Stem Cell Research and Therapy (H: 22), OA and Cartilage (H: 20), Biomaterials (H: 15), International Journal of Molecular Sciences (H: 15), Plos One (H: 14), American Journal of Sports Medicine (H: 13), Scientific Reports (H: 13), Arthritis Research and Therapy (H: 11), Journal of Orthopedic Research (H: 11), and Stem Cells International (H: 11).

### 3.3. Authors

**Figure 4:**
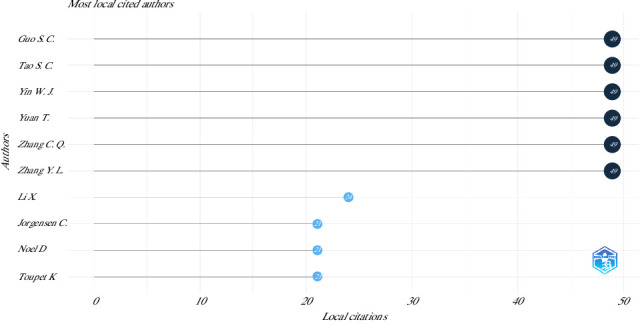
Top 10 most local cited authors on the use of mesenchymal stem cells in animal models of osteoarthritis (2015–2024).

The top 10 most relevant authors in this topic were Wang Y (35 articles), Wang J (33 articles), Zhang J [33] articles, Zhang X (31 articles), Liu Y (30 articles), Li X (29 articles), Li Y (29 articles), Wang X (29 articles), Zhang Z (29 articles), and Chen J (28 articles). On the other hand, the most local cited authors are shown in Figure 4.

**Figure 5:**
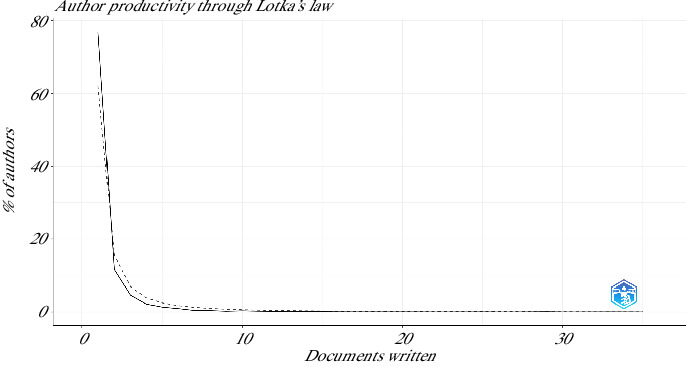
Authors' article production according to Lotka's law for the use of mesenchymal stem cells in animal models of osteoarthritis (2015–2024).

**Figure 6:**
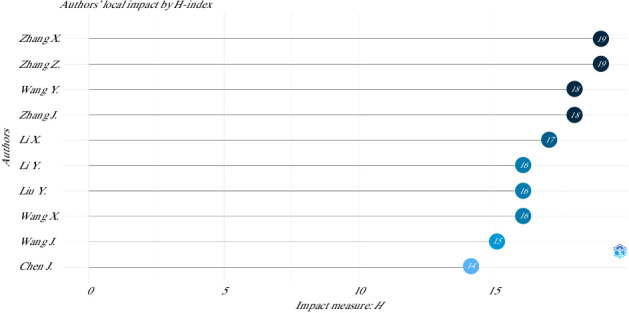
Top 10 authors' local impact on the use of mesenchymal stem cells in animal models of osteoarthritis (2015–2024).

An analysis of author productivity in this field, based on Lotka's law, which states that the number of authors publishing a given number of papers is inversely proportional to the number of papers published [35], revealed distinct patterns. Specifically, 77% (4101 authors) published only one paper, while 12% (618 authors) published two papers. The pattern continues with fewer authors publishing larger quantities: 2 authors each published between 17 and 27 articles, and 1 author published 35 articles (Figure 5). On the other hand, the local impact of the authors measured in terms of H-index is presented in Figure 6.

### 3.4. Institutions

The top 10 most relevant institutions related to the production of articles on this research topic were Zhejiang University (China) (153 articles), Shanghai Jiao Tong University (China) (108 articles), Peking University (China) (82 articles), Sichuan University (China) (82 articles), Southern Medical University (China) (82 articles), Sun Yat-Sen (China) (70 articles), Fourth Military Medical University (China) (57 articles), Tokyo Medical and Dental University (Japan) (56 articles), Soochow University (Taiwan) (53 articles), and Nanjing Medical University (China) (49 articles).

### 3.5. Countries

**Figure 7:**
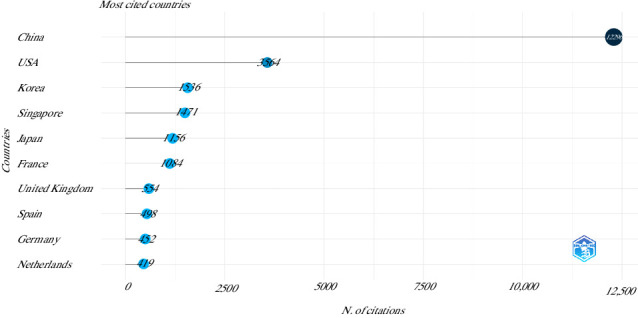
Top 10 most cited countries on the use of mesenchymal stem cells in animal models of osteoarthritis (2015–2024).

The top 10 most productive countries for articles on the use of MSCs in animal models of OA between 2015 and 2024 were China (481 articles), the USA (133 articles), South Korea (60 articles), Japan (57 articles), Spain (27 articles), France (25 articles), the United Kingdom (21 articles), Germany (20 articles), Iran (17 articles), and the Netherlands (17 articles). On the other hand, the most cited countries are shown in Figure 7.

### 3.6. Documents, References, and Cocitation Network

**Figure 8:**
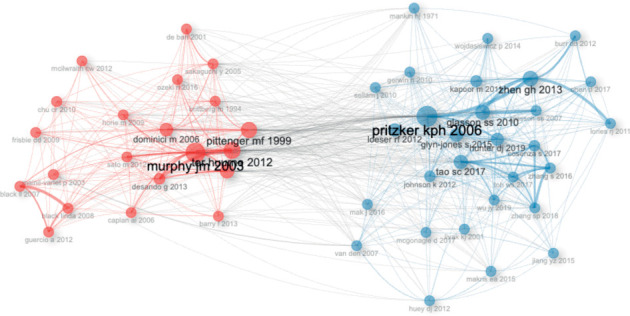
The cocitation network of the main references used in the articles on the use of mesenchymal stem cells in animal models of osteoarthritis (2015–2024).

The top 10 most globally cited articles according to the H-index are shown in Table 2. On the other hand, Table 3 shows the top 10 most cited references of the 1000 articles produced in the field of this bibliometric analysis. Regarding the cocitation network of the references used in the articles of this bibliometric analysis, two main interconnected clusters were identified; one of them mainly influenced by the papers was first authored by Glasson (USA) [36], Hunter (Australia) [37], and Pritzker (Canada) [38], and the other cluster by Murphy (USA) [39] (Figure 8).

### 3.7. Keywords

**Figure 9:**
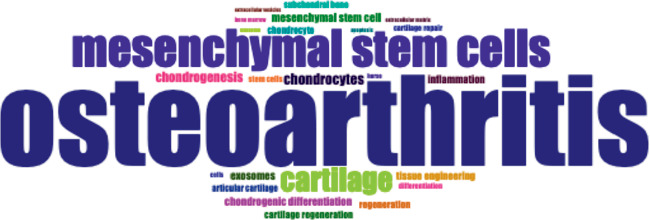
Top 25 most used keywords in the articles on the use of mesenchymal stem cells in animal models of osteoarthritis (2015–2024).

The top 10 most frequently used author keywords were OA (396 occurrences), MSCs (162 occurrences), cartilage (104 occurrences), chondrocytes (49 occurrences), chondrogenesis (45 occurrences), MSC (39 occurrences), chondrogenic differentiation (37 occurrences), exosomes (37 occurrences), inflammation (36 occurrences), and tissue engineering (35 occurrences). A cloud map of the top 25 most used author keywords in this bibliometric analysis is shown in Figure 9.

**Figure 10:**
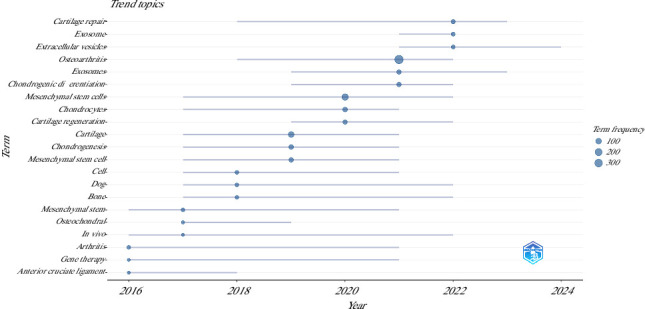
Trend topics in author keywords over time in the articles on the use of mesenchymal stem cells in animal models of osteoarthritis (2015–2024).

The analysis of the occurrences of author keywords over time (2015–2024) allowed to stablish the trend topics related to the three most frequent keywords per year considering a minimum of 10 occurrences per item (Figure 10).

### 3.8. Clustering

Clustering by coupling, considering the articles as the unit of analysis, measuring the coupling by references and considering the impact measure by local citation score, and labeling the clusters by author keywords revealed the existence of two large clusters of trending topics around which article production has been focused throughout observation (2015–2024).

**Figure 11:**
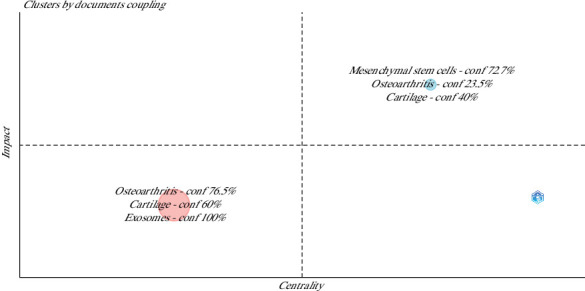
Clustering by coupling analysis of the author keywords in the articles on the use of mesenchymal stem cells in animal models of osteoarthritis (2015–2024). conf = confluence.

**Figure 12:**
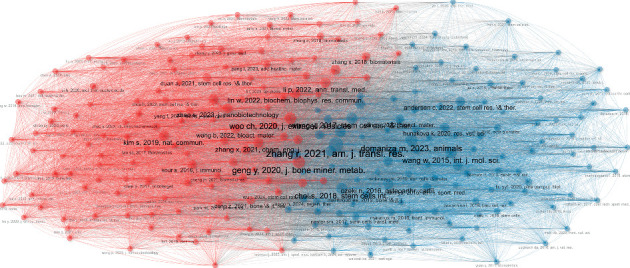
Clustering by coupling analysis of the articles coupled by the references and marked by author keywords on the articles on the use of mesenchymal stem cells in animal models of osteoarthritis (2015–2024). conf = confluence.

Cluster 1 was composed of “OA, cartilage, and exosomes,” while Cluster 2 is composed of “MSCs, OA, and cartilage” (Figure 11). On the other hand, Figure 12 shows how authors, references, and sources are arranged around these two great clusters.

### 3.9. Conceptual Structure

**Figure 13:**
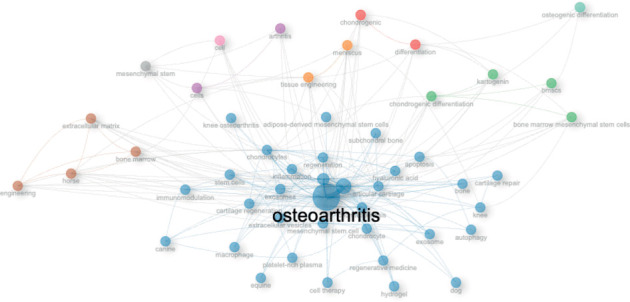
The co-occurrence network plot showing the most relevant clusters composed by author keywords and how they are interconnected in the articles on the use of mesenchymal stem cells in animal models of osteoarthritis (2015–2024).

Co-occurrence network analysis of the author keywords used in the articles on this topic revealed four clusters. Of note, Cluster 1 (in green) was the greatest cluster including clusters in which this research topic has grown and connected across publications during the observation period, not the details of the documents. Co-occurrence network analysis of author keywords revealed four main clusters. Cluster 1 (green) was the largest and represented the core thematic structure of the field, showing how this research topic has expanded and interconnected across publications during the observation period. This cluster included keywords such as OA, cartilage, and mesenchymal stem cells, among others. Cluster 1 showed multiple links with the smaller clusters (Clusters 2 [blue], 3 [red], and 4 [violet], while Cluster 5 (yellow) was connected exclusively to Cluster 2 (Figure 13).

**Figure 14:**
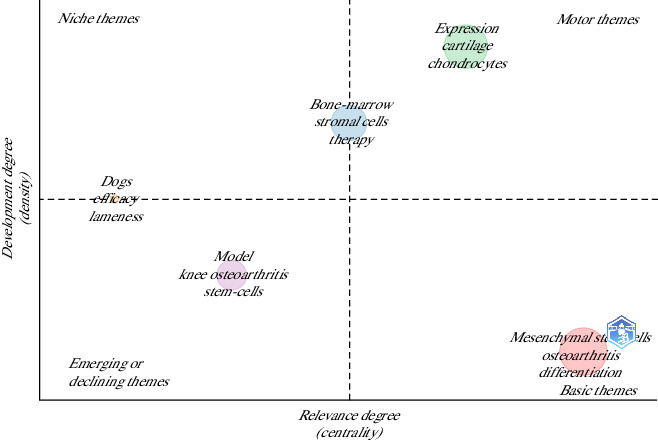
Thematic map showing the most relevant clusters of research themes according to the author keywords of the articles on the use of mesenchymal stem cells in animal models of osteoarthritis (2015–2024).

In general terms, the importance of the research trends (considering the author keywords) in this topic by evaluating the position of the clusters in a theme quadrant plot; 5 clusters were identified in order of relevance for their closeness to the center of the graph (Figure 14).

### 3.10. Social Structure

**Figure 15:**
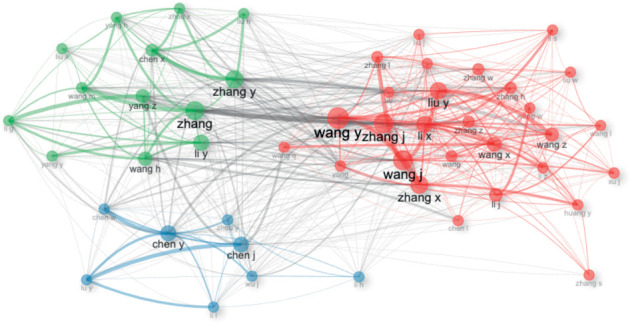
Collaboration network for authors.

**Figure 16:**
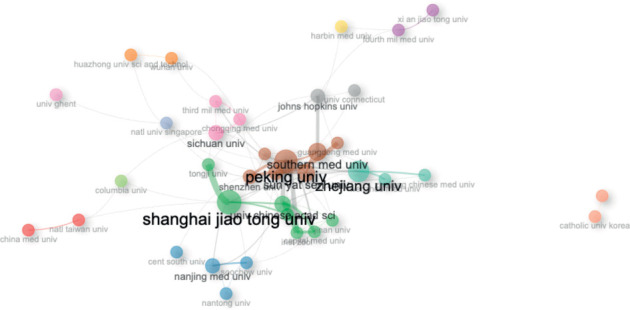
Collaboration network for institutions.

**Figure 17:**
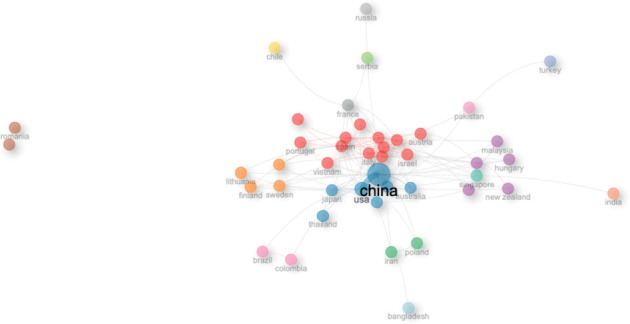
Collaboration network for countries.

The main network of authors (top 50) on this topic consists of three clusters, mainly led by Wan J, Chen Y, and Zhang, which show a high degree of interconnectedness (Figure 15). On the other hand, the main network of institutions is led by Chinese universities (Zhejiang University, Shanghai Jiao Tong University, and Peking University), which are interconnected with other institutions in China, the United States, Taiwan, Singapore, and Belgian institutions (Figure 16). In terms of the main network of countries, the United States of America and China agglutinate the interconnection with countries from Europe, the Midwest, and the Caribbean. It is noteworthy that isolated research has been conducted between Romania and Greece (Figure 17).

## 4. Discussion

According to the literature search, there are several published bibliometric analyses on the role of stem cells in cardiovascular [56, 57] and cerebrovascular diseases [58], plastic and reconstructive surgery [59], acute lung injury [60], and liver diseases [61], while one scientometric study has been published evaluating the role of these cells in meniscal regeneration [62]. In general, some of these studies presented observation periods of 10–20 years and mainly performed the bibliometric analyses using a combination of two (VOSviewer and CiteSpace) [61, 62] or three software packages (VOSviewer, CiteSpace, and Bibliometrix) [57, 60] or using only one software package (CiteSpace) [59]. The number of published bibliometric studies, as well as their observation periods, places stem cells as a trending topic in the field of regenerative medicine in several medical specialties.

Our results in terms of scientific production of article citations over the last 10 years were about 80% less than the scientific production related to the use of MSCs in cardiovascular diseases [57], 50% less than that in the field of liver diseases [61], almost similar in the field of ischemic stroke [58], and high when compared to the topics of acute lung injury [60], plastic surgery [59], and meniscal regeneration [62]. We included this comparison to place the bibliometric indicators of MSC-based therapies for OA in animal models within the wider context of stem cell research in other medical fields, thereby helping readers interpret the maturity and potential growth of this topic.

As mentioned above, according to Bradford's Law, the scientific production on this topic is concentrated in 19 sources. Specifically, this law postulates that when a particular topic is studied extensively, the number of journals publishing articles on that topic follows a predictable pattern. Thus, the distribution of articles is such that a small number of journals will publish the majority of articles (core journals), while a larger number of journals will publish fewer articles [34, 63]. Regarding the most productive sources found in this bibliometric study, it is important to consider that specialized journals such as Stem Cell Research and Therapy, International Journal of Molecular Sciences, and Stem Cells International are among the top 10 journals not only for the publication of articles on this topic but also for the publication of documents on the use of stem cells in cardiovascular diseases [57], ischemic stroke [58], acute lung injury [60], liver diseases [61], and meniscal regeneration [62].

On the other hand, the journals discussed in the study presented H-index values ranging from 5 to 47, while the topic of this bibliometric study presented H-index values ranging from 11 to 22 for all journals. The H-index is a useful measure of the impact and productivity of authors, institutions, countries, and journals. Specifically, for a journal, the H-index is calculated based on the number of documents (*h*) that have been cited at least *h* times. Thus, this bibliometric measure reflects both the quantity of published documents and their citation impact, providing a comprehensive view of the overall impact of the studies published in the journal, rather than focusing solely on individual articles [64].

The top 10 authors identified in this study were all Chinese researchers, none of whom were involved in academic work outside the specific field of regenerative medicine. It is important to note that Chinese researchers and institutions are at the forefront of stem cell research applied to cardiovascular disease [57], ischemic stroke [58], and liver disease [61]. In contrast, Japan leads in academic production related to meniscus regeneration [62].

This comparative perspective was intentionally included to contextualize the bibliometric indicators of MSC-based therapies for OA in animal models within the broader landscape of stem cell research applied to other medical specialties. By doing so, we aim to highlight both the relative maturity and the growth potential of this field, allowing readers to better interpret the observed publication trends and citation patterns.

China, including its universities and research institutions, is a global leader not only in stem cell research applied to animal models of OA but also in a wide range of stem cell research in various medical specialties. However, the basic references cited in the articles published during the period covered by this bibliometric study come mainly from researchers based in Canada, the United States of America, Italy, Korea, and Australia. In particular, two main cocitation clusters are identified: Cluster 1, led by seminal papers by Glasson et al. [36], Hunter and Bierma-Zeinstra [37], and Pritzker et al. [38], and Cluster 2, led by foundational papers by Murphy et al. [39] and Pittenger et al. [51]. These clusters reflect significant networks of influential research in the field.

The cloud plot of author keywords from this study highlights the main focus areas of research on stem cells and their exosomes in animal models of OA. The analysis shows a strong emphasis on the evaluation of cartilage components, chondrogenic differentiation, apoptosis, and inflammation. In addition, the research trend indicated by the author's keywords point to the investigation of the paracrine effects of MSCs, specifically mediated by their extracellular exosomes and vesicles, in cartilage repair. On the other hand, the conceptual structure analysis of the author keywords revealed two clusters, one of them focused in assessing the effect of exosomes in osteoarthritic cartilage, and the other in evaluating the effect of MSCs in osteoarthritic cartilage.

This separation between “exosomes” and “MSCs” in the clustering analysis reflects how bibliographic coupling and keyword co-occurrence patterns capture distinct research trajectories within the field. Although exosomes are biologically derived from MSCs, the bibliometric patterns indicate that many studies investigate exosomes as a specific therapeutic strategy, often with unique methodological frameworks, reference bases, and targeted outcomes that differ from broader MSC-based interventions. Consequently, the algorithm groups them into separate clusters, highlighting the emergence of exosome-focused research as an independent yet complementary stream within regenerative medicine for OA.

The thematic map of research trends shows that the central cluster focuses on topics such as bone marrow, stromal cells, and therapy. In contrast, emerging themes include dogs, efficacy, and lameness. These findings suggest that stem cell research may be valuable in dogs, particularly as pets. Dogs share similar pathophysiological mechanisms in OA development as humans, making them a relevant model for studying stem cell therapies [2, 8]. In addition, this research could open up significant market opportunities for orthobiologics.

In terms of the social structure of the present study, Chinese authors, countries, and institutions are at the center of the research network. Chinese authors and institutions are highly interconnected with each other, and they are also connected with researchers, institutions, and countries in different regions of the world. This central role of China highlights its significant influence and collaborative ties in the global stem cell research landscape.

## Figures and Tables

**Table 1:** Annual production and average citations per year of articles on the use of mesenchymal stem cells in animal models of osteoarthritis (2015–2024).

Year	2015	2016	2017	2018	2019	2020	2021	2022	2023	2024
Annual scientific production	60	75	94	98	97	117	135	132	106	86
Mean citations per year	39.9	43.9	42.2	43.3	38.4	22.6	19.4	11.9	5.60	0.80

**Table 2:** Top 10 most globally cited documents on the use of mesenchymal stem cells in animal models of osteoarthritis (2015–2024).

Authors	Source	Topic	Country	TC	TCPY	NTC
Zhang et al., [40]	Biomaterials	Mesenchymal stem cells, cartilage, apoptosis, immune reactivity	Singapore	600	85.71	13.86
Tao et al., [41]	Theranostics	Mesenchymal stem cells, exosomes, cartilage, osteoarthritis	China	501	62.63	11.83
Zhang et al., [42]	Osteoarthritis and Cartilage	Embryonic mesenchymal stem cells, exosomes, osteochondral regeneration	Singapore	474	52.67	10.81
Cosenza et al., [43]	Scientific Reports	Mesenchymal stem cells, cartilage, exosomes	France	421	52.63	9.94
Zhang et al., [44]	Biomaterials	Mesenchymal stem cells, cartilage, exosomes, inflammation	Singapore	344	57.33	8.93
Wu et al., [45]	Biomaterials	Mesenchymal stem cells, cartilage, exosomes, mTOR inhibition	China	342	57.00	8.88
Mao et al., [46]	Stem Cell Research and Therapy	Mesenchymal stem cells, cartilage, exosomes, WNT5A inhibition	China	307	43.86	7.09
Zhu et al., [47]	Stem Cell Research and Therapy	Pluripotent stem cell-derived mesenchymal stem cells, exosomes, osteoarthritis	China	297	37.13	7.01
Liu et al., [48]	Cell Cycle	Mesenchymal stem cells, cartilage, exosomes, apoptosis inhibition	China	233	33.29	5.38
Wang et al., [49]	Stem Cell Research and Therapy	Embryonic mesenchymal stem cells, osteoarthritis, cartilage extracellular matrix	China	231	28.88	5.46

Abbreviations: NTC = normalized total citations, TC = total citations, and TCPY = total citations per year.

**Table 3:** Top 10 most cited references in the articles on the use of mesenchymal stem cells in animal models of osteoarthritis (2015–2024).

Authors	Source	Topic	Country	TC
Pritzker et al., [38]	Osteoarthritis and Cartilage	Osteoarthritis, cartilage, grading, histopathology	Canada	102
Zhen et al., [50]	Nature Medicine	TGF-β, mesenchymal stem cells, osteoarthritis	USA	79
Murphy et al., [39]	Arthritis and Rheumatology	Stem cell therapy, osteoarthritis, goat model	USA	78
Pittenger et al., [51]	Science	Adult human mesenchymal stem cells	USA	75
Dominici et al., [52]	Cytotherapy	Mesenchymal stromal cells, classification	Italy	65
Glasson et al., [36]	Osteoarthritis and Cartilage	Histopathology assessment, cartilage, mouse	USA	62
Ter Huurne et al., [53]	Arthritis and Rheumatism	Adipose-derived stem cells, osteoarthritis	NL	59
Jo et al., [54]	Stem Cells	Mesenchymal stem cells, knee osteoarthritis	Korea	57
Kapoor et al., [55]	Nature Reviews Rheumatology	Proinflammatory cytokines, osteoarthritis	Canada	56
Hunter and Bierma-Zeinstra [37]	The Lancet	Osteoarthritis pathophysiology	Australia	55

*Note:* NL = The Netherlands.

Abbreviation: TC = total citations.

## Data Availability

The datasets used and/or analyzed during the current study will be available from the corresponding author upon reasonable request.
